# Stable mercury isotopes as a decision-support tool for public health

**DOI:** 10.3389/fpubh.2026.1822247

**Published:** 2026-06-29

**Authors:** Arioene Vreedzaam, Firoz Abdoel Wahid, Maureen Y. Lichtveld, Wilco Zijlmans, Jeffrey Wickliffe

**Affiliations:** 1 Faculty of Medical Sciences, Anton de Kom University of Suriname, Paramaribo, Suriname; 2 Department of Environmental and Occupational Health, School of Public Health, University of Pittsburgh, Pittsburgh, PA, United States; 3 Department of Environmental Health Sciences, School of Public Health, University of Alabama at Birmingham, Birmingham, AL, United States

**Keywords:** ASGM, exposure sources and pathways, mercury stable isotopes, public health, Suriname

## Abstract

Widespread mercury exposure remains a critical global health challenge, particularly in regions affected by artisanal and small-scale gold mining (ASGM). Conventional biomonitoring documents “body burden,” it often fails to pinpoint specific environmental sources, hindering targeted public health interventions. This viewpoint article proposes reframing mercury stable isotope analysis as a decision-support tool within existing public health workflows. Using Suriname as an illustrative case, we demonstrate how isotopic “fingerprints” resolve complex exposure pathways that concentration data alone cannot. We provide a systematic three-step framework, from routine detection to source attribution and targeted intervention, enabling practitioners to design evidence-based, equitable strategies that align with international monitoring priorities under the Minamata Convention.

## Introduction: mercury exposure as a global health challenge

Mercury exposure represents an urgent global health challenge, persisting despite decades of research. As a global pollutant, it is a major concern in low- and middle-income countries (LMICs) and ASGM regions (1, 2). Particularly, its organic form methylmercury (MeHg) can bioaccumulate and biomagnify through aquatic food webs, often reaching high concentrations in fish (1). This disproportionately burdens fish-reliant Indigenous communities and vulnerable populations, such as pregnant women and children (3, 4). Exposure to methylmercury impairs neurological development, leading to lifelong cognitive deficits and intergenerational inequities (5).

While anthropogenic activities are central to mercury contamination, it is essential to account for geogenic Hg: naturally occurring Hg released through geological weathering and mineralization, which is particularly prevalent in the Amazonian regions and can complicate exposure baselines (6–9).

International frameworks like the Minamata Convention emphasize monitoring, but a persistent challenge remains: the inability to translate collected data into targeted action (10, 11). Current surveillance systems document “body burden” but only complete a portion of the exposure pathway. Accurately identifying environmental sources is critical for designing interventions that eliminate hazardous exposures. Without forensic precision, public health efforts remain reactive rather than preventive.

## Limitations of current mercury surveillance methods

Current programs rely heavily on measuring total mercury concentrations in environmental media or biological samples (blood, hair, urine). Although essential for risk calculation, these measurements typically fail to pinpoint the original source of mercury. This lack of source attribution is problematic when multiple exposure pathways overlap. For example, total mercury measurements cannot distinguish whether exposure is driven by dietary fish, localized ASGM contamination, or mercury-containing personal care products.

Without the ability to differentiate between geogenic and anthropogenic releases, public health responses risk being misdirected. This gap makes it difficult to evaluate policy effectiveness or target remediation toward the correct behaviors. Furthermore, the disconnect between documentation and source identification leads to tangible societal costs and persistent inequities. This gap persists because advanced sourcing methods are often perceived as too costly or technically demanding for LMICs.

## Mercury stable isotopes as a public health intervention

Mercury stable isotope analysis (SIA) should be viewed as a practical decision-support tool for public health surveillance, rather than a niche technique reserved for environmental scientists. In regions impacted by artisanal and small-scale gold mining (ASGM) or other contamination sources, practitioners often lack the forensic tools to identify the specific origin of exposure, which limits their ability to design targeted and effective interventions.

This approach leverages two complementary isotopic signatures to resolve exposure pathways that total mercury concentrations alone cannot. Mass-dependent fractionation (MDF; reported as *δ*^202^Hg) reflects biogeochemical processing, while Mass-independent fractionation (MIF; reported as Δ^199^Hg and Δ^200^Hg) is particularly useful for distinguishing atmospheric from local sources (12–14). By tracing these distinct “fingerprints,” SIA answers the critical question of source origin, a method widely validated in environmental and human health studies (15–19).

**Figure 1:**
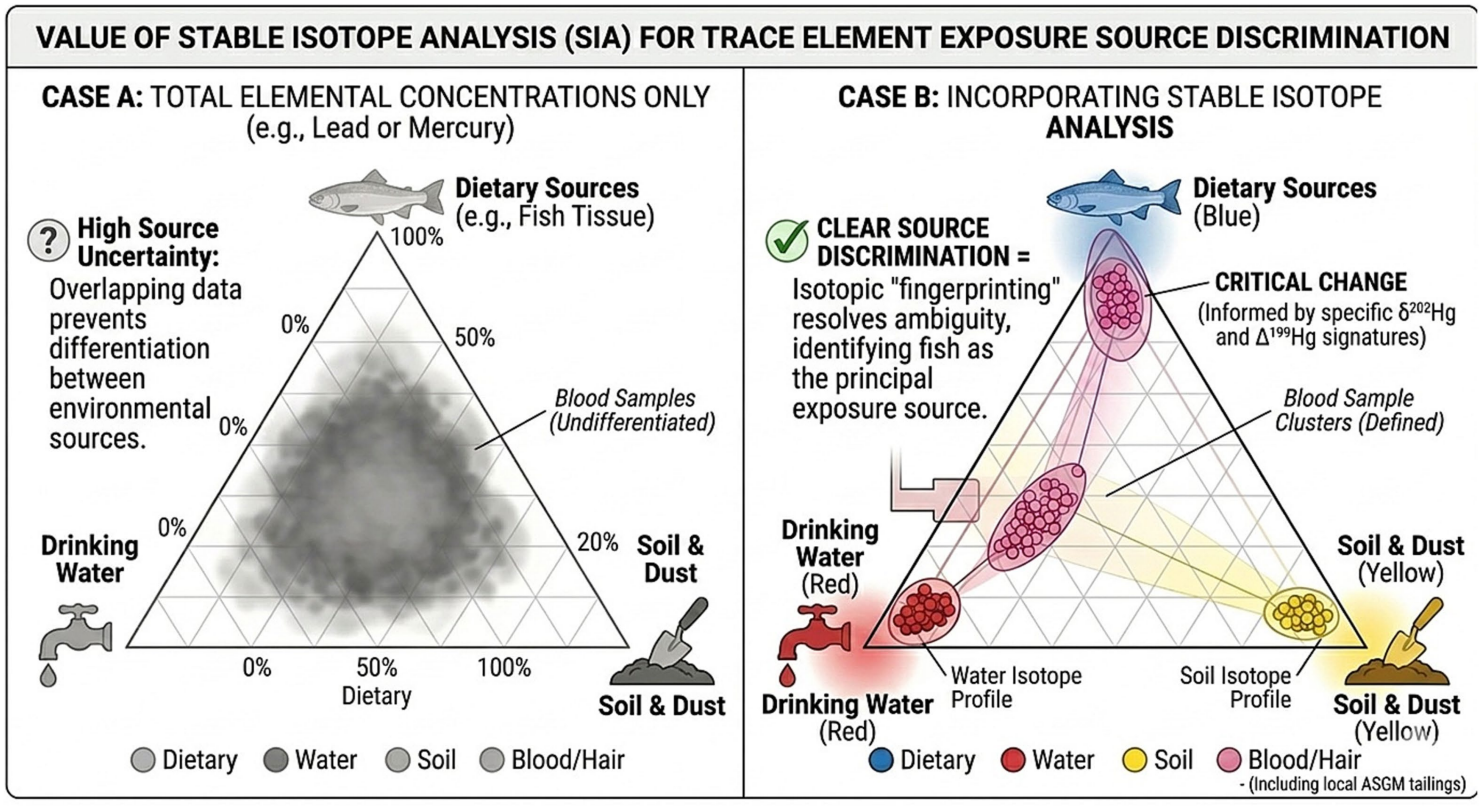
Cartoon representation illustrating the additional value that stable isotopes in conjunction with total element analysis offers in terms of discriminating sources of exposure in comparison to an approach using only a total element analysis which provides inadequate resolution.

Crucially, public health staff do not need deep technical expertise to benefit from this approach. Rather, success depends on awareness of the tool’s utility and established partnerships with academic or laboratory experts. Integrating SIA as a decision-support step within existing workflows enhances surveillance and interventions planning without imposing additional technical burdens. By leveraging these distinct isotopic “fingerprints,” we can resolve complex exposure pathways that traditional surveillance often leaves ambiguous (see Figure 1).

## Added value of stable isotope approaches for public health practice

Stable isotope analysis shifts the focus from measurement to actionable interpretation. By enabling source attribution, these approaches reduce uncertainty at key decision points. Isotope analysis can:

- Distinguish between dietary, mining-related, and broader environmental sources (19–22).
- Trace the origin and fate of methylmercury across environmental and biological matrices (16, 19, 23–25).
- Inform maternal and child health risk assessments in low-resourced settings (17, 18).
- Support more targeted intervention strategies and context-specific risk communication (5).

## An illustrative case: mercury exposure in Suriname

The application of mercury stable isotope analysis in Suriname illustrates how this approach can inform public health decision-making in settings characterized by ASGM activity, fish-based diets, and overlapping environmental, ecological and social determinants of health. Indigenous and Tribal populations in the affected regions of Suriname rely heavily on freshwater fish as a primary protein source, increasing vulnerability to methylmercury exposure through diet. In such contexts, total mercury measurements alone may be insufficient to disentangle dietary exposure from mining-related contamination or broader environmental sources.

While THg monitoring (hair & fish) has identified contamination “hotspots,” it cannot explain high mercury levels in areas remote from mining or anomalous burdens in specific fish species. For instance, proximity to mining and fish size are poor predictors of mercury levels; piscivorous fish exceeding global tropical averages accounted for 52% in background sites, 79% upstream of ASGM, and 90% downstream (26).

Isotopic analyses were performed on select piscivorous fish tissues and human hair. The fish species selected for sampling was based on diet preferences of community members. The results from the analysis transformed our understanding of the ambiguity (Figure 1, Case B). By analyzing δ^202^Hg (mass-dependent fractionation) and Δ^199^Hg (mass-independent fractionation) ratios across different river basins, two distinct contamination scenarios were identified.

- Direct ASGM impact regions: These isotopic “fingerprints” closely matched the liquid elemental mercury (Hg^0^) used in gold processing. Low (near-zero) Δ^199^Hg values indicate rapid transfer and local methylation with limited prior photochemical processing.
- Atmospheric deposition regions: In areas that were assumed as “pristine/background” high mercury levels were detected. ASGM-impacted fish exhibited Δ^199^Hg values near 0.0–0.2‰, whereas sites dominated by atmospheric deposition showed Δ^199^Hg values typically >0.5‰ (26). Δ^200^Hg, when available, provides an additional tracer of atmospheric Hg and will be recommended for future sampling (26).

Without stable isotope data, public health interventions might incorrectly prioritize local mining suppression for all affected regions. This evidence clarifies that while mining regulation is vital in ASGM areas, it would be ineffective in remote regions, where interventions must instead focus on dietary substitutions and international policy (e.g., the Minamata Convention). This distinction prevents the misallocation of resources and avoids unfairly penalizing communities for distal contamination. The “local mining vs. global atmosphere” contrast is presented as an illustrative example; the framework is intended to be flexible and applicable to other source categories (e.g., geogenic, consumer products, mixed sources).

## Implications for science, policy, and public health action

The application of mercury stable isotope analysis in public health contexts extends the use of this approach beyond source characterization toward hypothesis testing and longitudinal epidemiologic cohort studies ultimately leading to decision-relevant interpretation. It highlights the value of interdisciplinary science that links geochemical methods with epidemiology, exposure science, and applied public health research.

Stable isotope analysis can complement existing biomonitoring approaches by functioning as a referral or decision-support tool when elevated mercury levels are detected and source attribution is necessary to inform intervention. These analyses can be incorporated at key-decision points rather than applied universally. Public health agencies may, for instance, deploy isotope analysis in sentinel populations, in communities experiencing sudden changes in exposure patterns, or in settings where regulatory or remediation decisions hinge on identifying dominant sources of mercury. This targeted use aligns with international monitoring and reporting priorities under the Minamata Convention (27) and can support more focused regulatory and enforcement strategies by providing stronger evidence for source-specific accountability.

Isotope-informed insights also have practical value for risk communication. By clarifying dominant exposure pathways, public health practitioners can provide more nuanced, community-specific guidance that reflects local environmental and dietary contexts. This can improve the credibility and relevance of public health messaging, particularly in communities where mercury exposure is closely linked to livelihoods, food systems, or environmental change.

Finally, incorporating isotope analysis into surveillance systems can strengthen program evaluation and policy learning. Isotope data can help assess whether interventions and policy measures are effectively addressing dominant sources of exposure over time, rather than relying solely on changes in total mercury concentrations.

## Implementation and equity considerations

Practical implementation of isotope-informed surveillance requires attention to laboratory capacity, cost, and partnership models. Regional and international collaborations can play a key role in ensuring access to technical expertise while minimizing duplication of resources (28). Ethical considerations are also central, including governance of biomonitoring data and meaningful community engagement in the interpretation and use of findings.

An explicit equity lens is essential. Integrating advanced analytical tools into public health practice should enhance, rather than exacerbate, disparities. Capacity building and co-development with affected communities and local institutions are therefore integral components of implementation, ensuring that the benefits of improved exposure interpretation translate into tangible public health gains.

## Conclusion and call to action

Mercury stable isotope analysis offers a practical opportunity to bridge the gap between exposure assessment and public health action. By serving as a decision-support tool within existing workflows, isotope-informed approaches improve how mercury data are interpreted and communicated, particularly where multiple exposure pathways complicate intervention planning.

As a complement to routine biomonitoring, this analysis can be integrated selectively at key decision points. When practitioners face elevated mercury levels from an uncertain origin, the following three-step framework enables an efficient move from detection to targeted intervention:

1 Routine detection (the trigger). Utilize existing THg biomonitoring to identify “At-Risk” populations. This includes human hair or blood sampling (hair for longer term dietary exposure; blood for recent exposure) and THg measurement.
2 The black box (isotope analysis). Deploy isotope analysis in “high uncertainty” zones where the source (local mining vs. global atmosphere vs. geogenic) is unclear. Recommended sampling matrices: piscivorous and lower trophic fish tissues; suspended particulate matter (SPM); surface sediments; soil and tailings near mining sites.

- Isotope priorities: δ^202^Hg (MDF) and Δ^199^Hg (MIF) for most source attribution questions; include Δ^200^Hg when atmospheric vs. local partitioning is a key decision point. Isotope ratios provide a distinct “fingerprint” for geogenic sources that differs from the signature of elemental mercury used in ASGM.

3 Targeted intervention (Table 1: intervention toolkit).

**Table 1:** Intervention toolkit.

Identified source	Practitioner action (the intervention)	Policy link
Dietary (fish)	Context-specific fish consumption advisories; promoting lower-mercury protein alternatives.	Strengthening food safety standards and local fishing regulations.
Mining (ASGM)	Occupational health training for miners; environmental remediation of tailings; dust control.	Aligning with National Action Plans (NAPs) under the Minamata Convention.
Consumer products	Targeted market “buy-back” programs; public awareness campaigns on toxic cosmetics.	Enforcement of import bans on mercury-added products (Art. 4 of Minamata).
Geogenic (natural)	Long-term monitoring of baseline levels; targeted dietary advisories for populations in high mineralization zones.	Establishing long-term ecological baselines; integrating geological data into public health planning.

By following this logic, practitioners can distinguish between dietary guidance, environmental remediation, regulatory action, or occupational risk reduction. For instance, if the isotope “fingerprint” identifies a dietary source, the intervention focuses on context-specific food advisories; if it identifies artisanal mining, the focus shifts to occupational safety or environmental remediation; and if it identifies personal care products, the practitioner can trigger regulatory enforcement.

Advancing this approach will require collaboration among researchers, public health agencies, and global health institutions to pilot and refine implementation models in high-risk settings. Priority actions include building analytical capacity through regional partnerships, developing guidance on when and how isotope analysis should be applied in surveillance and response frameworks, and ensuring alignment with international monitoring and reporting efforts. Such collaboration can support approaches across diverse geographic and institutional contexts.

By improving the interpretability and actionability of mercury exposure data, the integration of stable isotope analysis into public health practice has the potential to enhance intervention effectiveness and promote more equitable responses to mercury exposure. As global efforts to reduce mercury-related health risks continue, incorporating decision-support tools that clarify exposure pathways can strengthen public health systems’ ability to protect vulnerable populations and respond to environmental change. In this context, the strategic integration of mercury stable isotope analysis into public health surveillance and response frameworks can support the objectives of the Minamata Convention by strengthening evidence-based decision-making and enabling more targeted, equitable interventions to reduce mercury-related health risks.

## Data Availability

Publicly available datasets were analyzed in this study. This data can be found at: https://doi.org/10.1016/j.envpol.2023.122447.
